# A study on the influences of a hygrothermal environment on the compressive strength and failure criteria of asphalt mixtures based on true triaxial tests

**DOI:** 10.1016/j.heliyon.2022.e10060

**Published:** 2022-08-01

**Authors:** Pan Wang, Mohamed Elchalakani, Yiming Zhou, Shi-tao Yan, Shuang-bei Li

**Affiliations:** a College of Civil Engineering and Architecture, Guangxi University, Nanning, 530004, China; b Key Laboratory of Disaster Prevention and Structural Safety of the Education Ministry, Guangxi University, Nanning, 530004, China; c School of Engineering, Department of Civil, Environmental and Mining Engineering, University of Western Australia, 35 Stirling Hwy, Crawley, 6009, WA, Australia

**Keywords:** Asphalt mixture, Damp-heat coupling, True triaxial test, Strength theory, Failure criteria

## Abstract

Based on a high-pressure servo static and dynamic true triaxial test machine (tawz-500/300), uniaxial and true triaxial tests of AC-25C asphalt mixtures under different heat moisture coupling treatments were performed, and the triaxial compressive strength value was determined by mathematical treatment. The test results show that in the range of 20–60 °C, the uniaxial and triaxial compressive strength of the AC-25C asphalt mixture decreases with increasing drying and soaking environment temperature. In the temperature range of 20–40 °C, the drying temperature sensitivity and soaking temperature sensitivity of the asphalt mixture with compressive strength and failure strain value as indices are the maximum. The increase in the intermediate principal stress can improve the triaxial compressive strength, and the increase reaches the maximum when the environmental treatment temperature is 40 °C. The maximum stress ratio is in the range of σ_2_/σ_3_ = 0.25 to σ_2_/σ_3_ = 0.5. The failure forms of uniaxial tension and triaxial tension are mainly caused by tensile stress. The influence of temperature, humidity and stress ratio on triaxial failure strength is analysed. The relationship between the failure strength and temperature coefficient k is established using a variety of failure criteria, which can provide an experimental and theoretical basis for the mechanical analysis of asphalt mixtures under complex stress states.

## 1 Introduction

The asphalt mixture shows mechanical properties under different temperatures and humidities [1, 2]. Pan [3] took the Nanyou expressway in Guangxi, China as an example and studied the influence of different factors, such as temperature, rainfall and load, on the service performance of asphalt pavement by means of multifactor analysis of variance. The study shows that the hygrothermal environment causes great damage to the service life of asphalt pavement. Roads in South China are under hygrothermal environments, but there are still some limitations in the use and design of pavement performance under this environment in China's current specifications [4]. Therefore, the influence of temperature and humidity on the mechanical properties of asphalt mixtures has always received broad attention among scholars. However, most studies focus on the mechanical properties of asphalt mixtures under room temperature and low-temperature environments, and studies on the influence of hygrothermal environments on asphalt mixtures are still limited [5, 6]. Based on the finite element method, Li [7] studied the thermal hydraulic mechanical response of pavement under vehicle load and established a thermos-hydro-mechanical model (THMM). Thus, we conducted experimental studies on the mechanical properties of asphalt mixtures in a hygrothermal environment.

Generally, it is difficult to use analytic methods to investigate the mechanical properties of asphalt mixtures owing to heterogeneity. Therefore, scholars increasingly use experimental methods to obtain the mechanical properties of asphalt mixtures. The field sampling test can truly reflect the actual pavement mechanical response level. Mabrouk [8] analyzed the use of pavement structure model and traffic speed deflector, and the results of field test were used in the modeling process. For the specimens sampled on site, the influence of aging and reducing the performance and failure strength of asphalt mixture relative to the control and original state is inevitable. Some researchers have studied the effect of aging of asphalt mixtures, bituminous materials, the effects of mixtures made of RAP and aged materials [9, 10, 11, 12]. Among many experimental methods, triaxial tests are widely used because they can accurately simulate mechanical conditions [13, 14]. Currently, triaxial tests are used to study brittle materials such as concrete and rocks; thus, reports about triaxial tests on asphalt mixtures are very limited [15]. Currently, split tensile tests and flexural tests based on a single indicator are most commonly used for asphalt mixtures [16, 17]. In fact, the stress direction of an asphalt mixture is not unique in actual situations. In recent years, some scholars have started to use triaxial tests to study the mechanical properties of asphalt mixtures [18]. Man et al. [19] studied the three-dimensional (3D) mechanical response of multi-layer transverse isotropic structure of pavement structure under moving vehicle load based on spectral element technology. Rahmani et al. [20] measured the creep characteristics of asphalt concrete by conventional triaxial static load tests and repeated load tests. Wang et al. [21] studied the relationship between the anisotropy and modulus of asphalt mixtures by performing triaxial loading tests. There are many studies on the mechanical properties of asphalt concrete for hydraulic structure core walls by conventional triaxial tests [22, 23, 24]. Zheng and Huang [25] studied the triaxial failure characteristics of asphalt concrete through a special form of confining pressure triaxial test and established a three-dimensional failure criterion. However, the conventional triaxial test can only exert axial pressure and confining pressure on the specimen and cannot realize triaxial loading, which means that the influence of intermediate principal stress on the strength of asphalt concrete is not considered [26, 27].

Other major failure mode and degradation mechanism for the asphalt mixtures are "freeze/thaw cycle", "low and intermediate temperature cracking", "moisture susceptibility" etc. In cold regions, asphalt concrete cracking caused by low temperature is known as the most important type of damage to asphaltic surfaces pavements [28, 29]. Temperature increase resulted in decreasing fracture energy mainly due to bond weakening in the chemical structure of bitumen [30]. The improvement of asphalt matrix material can effectively improve the performance of asphalt mixture, such as cracking resistance at low temperature and rutting resistance at high temperature [31, 32].

The heterogeneity of asphalt mixture can affect the strength properties and failure behavior and fracture path in these randomly distributed composite materials. Aliha et al. [33] considered the non-uniformity of asphalt mixture in the research and modeling, and improved the position of pressure intensity factor. The dimensional finite element analyses show that the geometry factors are not sensitive to the Poisson's ratios of aggregates and mastic [34]. He et al. [35] studied the fracture propagation trajectory of heterogeneous asphalt composites. Compared to brittle and isotropic materials, the fracture path of the asphalt mixture shows more deviation, and this deviation increases for those mixtures containing coarser aggregates in the ligament and tested under medium temperature conditions.

Guan et al. [36] studied the intermediate stress effect of asphalt mixtures at low temperature using a homemade true triaxial apparatus; they pointed out that the intermediate principal stress had a significant impact on the strength of asphalt mixtures; the authors also studied the impact of various factors on the strength of asphalt concrete under true triaxial stress. Yang et al. [37] proposed that the intermediate principal stress can be used as an important assessment factor of the strength of asphalt concrete under low temperature. Wu [38] evaluated the influence of various parameters on the performance of asphalt mixtures under high temperature through triaxial tests and found that intermediate principal stress should be considered when the mechanical properties of asphalt mixtures were studied.

Based on the abovementioned studies, researchers have performed research on the triaxial stress strength criterion of asphalt concrete. Huang et al. [39, 40, 41, 42] proposed failure criteria based on the octahedral stress space under true triaxial loading, but transverse loading was not considered in the experiments. Suo [43] determined the elastic limit of asphalt concrete based on the true triaxial test in the field of on-line elasticity. Guan et al. [36] established the true triaxial strength equation of SBS-modified asphalt AC-13C and SUP-13C asphalt mixtures and applied it to the verification of low-temperature bending tests and pavement cracking point prediction. The abovementioned studies promoted the development of the strength theory of asphalt concrete under triaxial stress and achieved some results. However, there are few reports on the mechanical properties and strength theory of asphalt concrete under hygrothermal environments. Some abovementioned studies have indicated that the intermediate principal stress, which is a significant factor for the mechanical properties of asphalt concrete, is closely associated with temperature and humidity. Therefore, it is essential and meaningful to conduct studies on the performance of asphalt concrete in hygrothermal environments.

The objective of this study is to evaluate the effects of the complex stress states and hygrothermal environments in asphalt mixtures in the laboratory, as well as to perform true triaxial tests for AC-25 C asphalt mixtures under hygrothermal environments. In addition, nonlinear and linear failure criteria under three-dimensional stress states are established to evaluate the impact of stress and hygrothermal environments on the performance of these asphalt mixtures. This study, for the first time, proposed a strength criterion of asphalt concrete under hygrothermal environments considering influencing factor *k*, which can predict the failure trend of asphalt mixtures in a certain range and provide suggestions and references for engineering applications.

## 2 Materials and methods

### 2.1 Materials and sample preparation

Marshall or rutting plate specimens are mostly used in the existing research on asphalt mixtures. The specimen size is small, which makes it difficult to meet the requirements of a true triaxial tester. In this study, the test specimens are taken from the test section of the lower layer of the AC-25C graded asphalt mixture of the Chongzuo Shuikou expressway. By cutting and sampling the qualified test section, the test piece meeting the size requirements of the tester can be obtained, which is conducive to reducing the size effect of the test piece and improving the compaction level. During the construction of asphalt pavement, the construction should be performed in strict accordance with the technical code for on-site construction of highway asphalt pavement (JTG F40-2004) and the code for design of highway asphalt pavement (JTG d50-2017). The test results can better represent the actual project.

**Figure 1:**
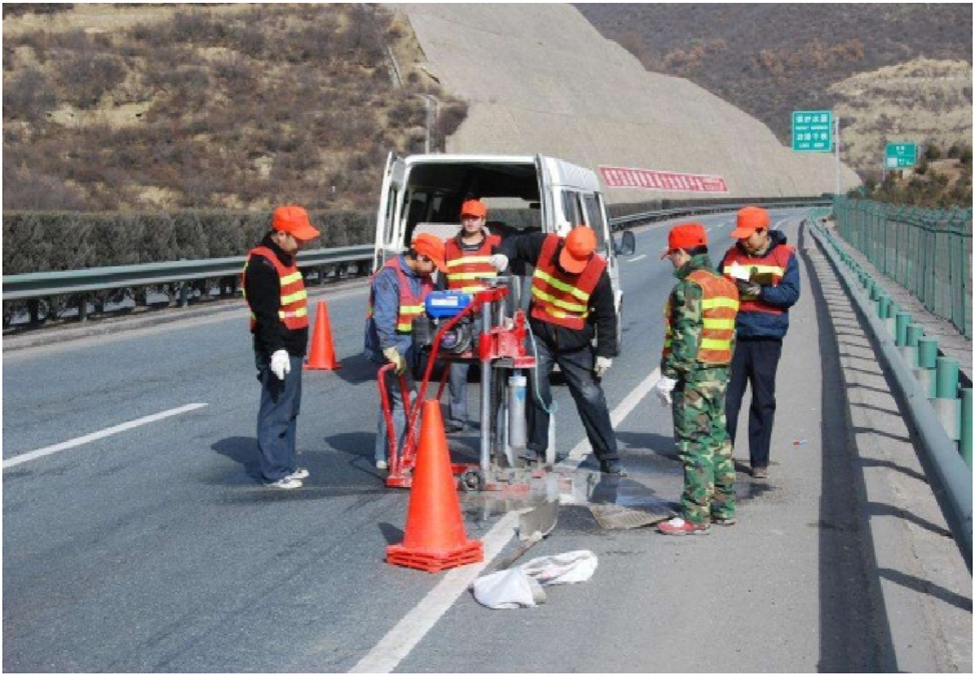
Field sampling.

**Figure 2:**
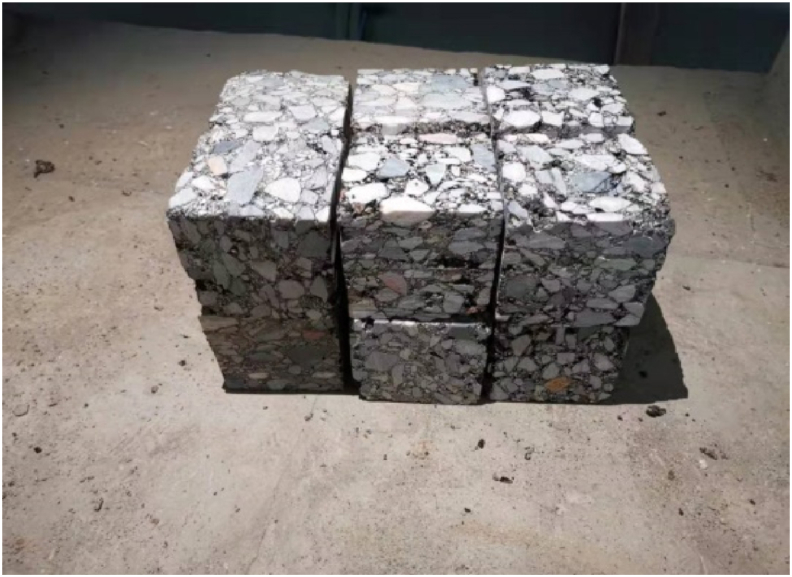
Asphalt concrete samples.

The test piece used in the test is a cube, and the size of the cut test piece on site is 100 mm × 100 mm × 100 mm. After the test piece was transported back to the laboratory, it was cut by an infrared cutting machine, and the six planes of the test piece were polished by an end grinding machine (Figures 1 and 2). After grinding, the effective size of the control specimen is 80 mm × 80 mm × 80 mm, and the specified dimensional error is ±2 mm. The material performance grade and permeability grade, aggregate type, aggregate particle size, aggregate gradation and mix proportion design are shown in Table 1, 2, 3, 4.

**Table 1:** Technical index of base asphalt.

Index	Unit	Design requirement	Inspection results	Single evaluation
Penetration	0.1mm	60–70	61	qualified
Penetration index PI	—	-1.5– ± 1.0	-1.124	qualified
Softening point	°C	≥47	49.5	qualified
10 °C ductility	Cm	—	22	qualified
15 °C ductility	Cm	≥100	＞100	qualified
Wax content (distillation method)	%	≤2.0	1.8	qualified
60 °C dynamic viscosity	Pa∗s	≥200	216	qualified
Flash point	°C	≥260	287	qualified
Solubility	%	≥99.5	99.7	qualified
Density (15 °C)	g/cm^3^	measured record	1.033	qualified
Quality change	%	± 0.6	-0.093	qualified
Residual penetration ratio (25 °C)	%	≥63	70	qualified
Residual ductility (10 °C)	Cm	≥6	8	qualified

**Table 2:** Technical index of basalt aggregate.

Aggregate size	Proportion (%)	Gross bulk density	Apparent density
22–30 mm	17	2.856	2.928
16–22 mm	19.5	2.849	2.917
11–16 mm	17	2.843	2.917
7–11 mm	14	2.837	2.915
4–7 mm	7	2.825	2.910
0–4 mm	21.5	2.818	2.887

**Table 3:** Mineral aggregate ratio and oilstone ratio.

Proportion of various mineral materials (%)								Oil stone ratio (%)
/	1#	2#	3#	4#	5#	6#	Mineral powder	
Target grading	21.0	16.5	16.0	16.0	6.0	24.0	0.5	3.9
Production grading	17	19.5	17	14	7	24.5	4	3.8

**Table 4:** Target and production mix proportion.

	Mass percentage passing the following sieve holes (square sieve, mm) (%)												
Gradation sieve	31.5	26.5	19.0	16.0	13.2	9.5	4.75	2.36	1.18	0.6	0.3	0.15	0.075
Target grading	100	97.4	79.7	67.6	57.0	46.2	32.1	22.6	16.1	11.5	7.8	6.3	5.0
Production grading	100	99.5	81.4	71.1	57.6	45.6	27.6	23.5	18.1	13.3	8.9	5.9	4.5

### 2.2 Experimental design and simulation of the hygrothermal environment

**Figure 3:**
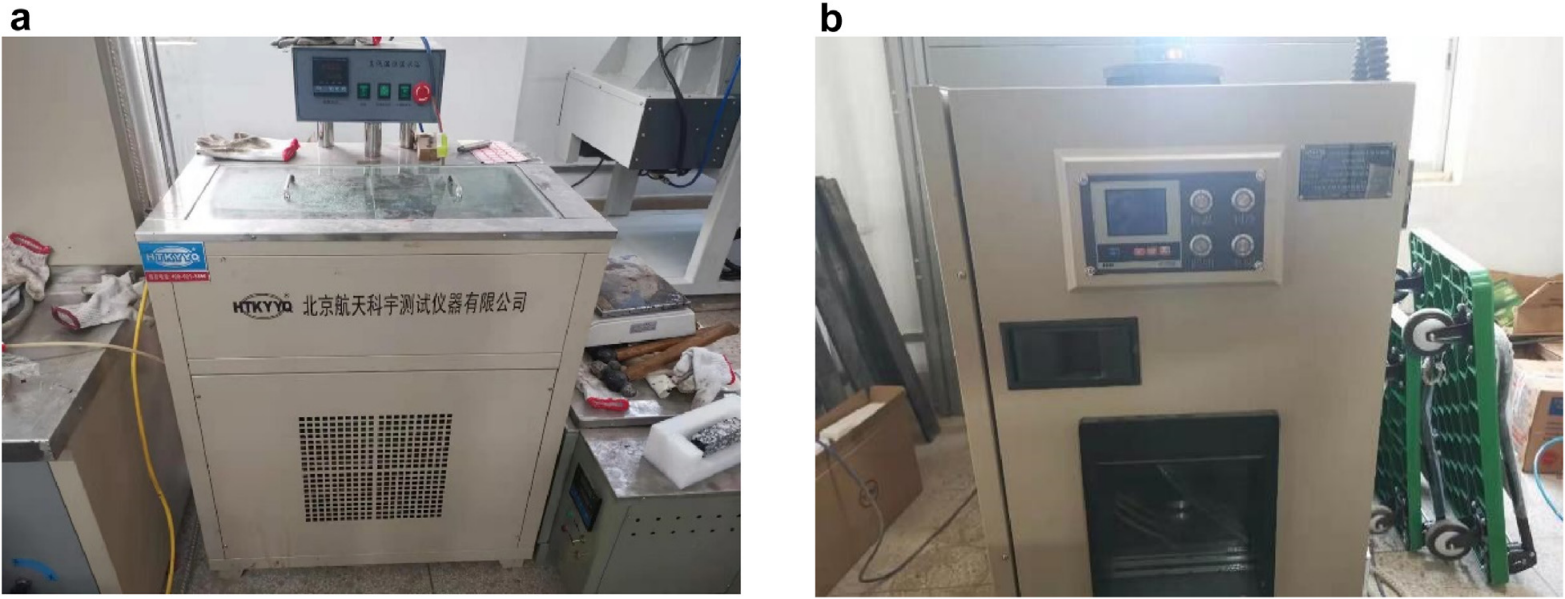
(a) constant temperature water bath cabinet and (b) constant temperature drying chamber.

An asphalt mixture is a viscoelastic–plastic material, and its strength greatly varies with changes in temperature and humidity. Therefore, the temperatures and humidity in the experiments need to be set according to the real environments to make the results consistent with the actual situation. Pan et al. [3] found that there is a certain hysteresis in the transmission of external air temperature to the interior of asphalt pavement through long-term temperature monitoring of each structural layer of asphalt pavement of an expressway in Nanning, Guangxi, and the temperature of asphalt pavement generally fluctuates in the range of 20–60 °C. The temperature of asphalt pavement generally fluctuates in the range of 20–40 °C on rainy days. Considering the abovementioned characteristics of asphalt concrete pavements in South China, the conditions of the samples were set as follows: 20 °C immersion, 20 °C dried, 40 °C immersion, 40 °C dried, 60 °C immersion and 60 °C dried. The immersion condition of the samples was achieved by a constant temperature water bath cabinet as shown in Figure 3(a); the dried condition of the samples was achieved by a constant temperature drying chamber for 24 h as shown in Figure 3(b). The experiments were performed immediately after removing the samples from the constant temperature water bath cabinet and constant temperature drying chamber. The temperature was controlled by an air conditioner during the experiments.

**Figure 4:**
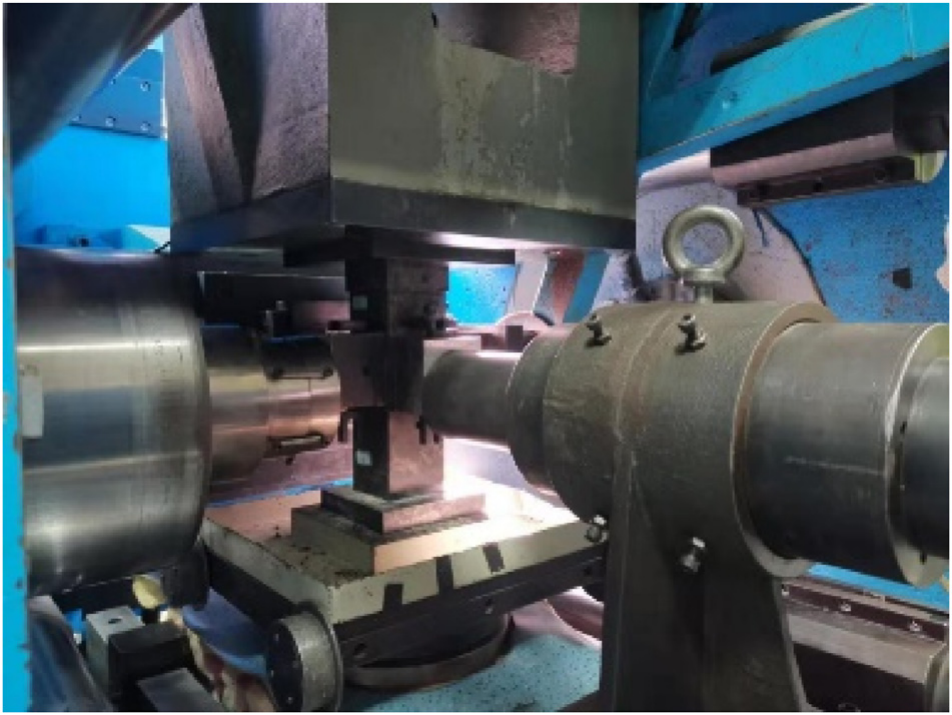
True triaxial compression test.

We used a high-pressure servo static and dynamic true triaxial testing machine for uniaxial and true triaxial compression loading (as shown in Figure 4). The stress and strain data during the experiment were collected by the sensors on the true triaxial testing machine.

**Figure 5:**
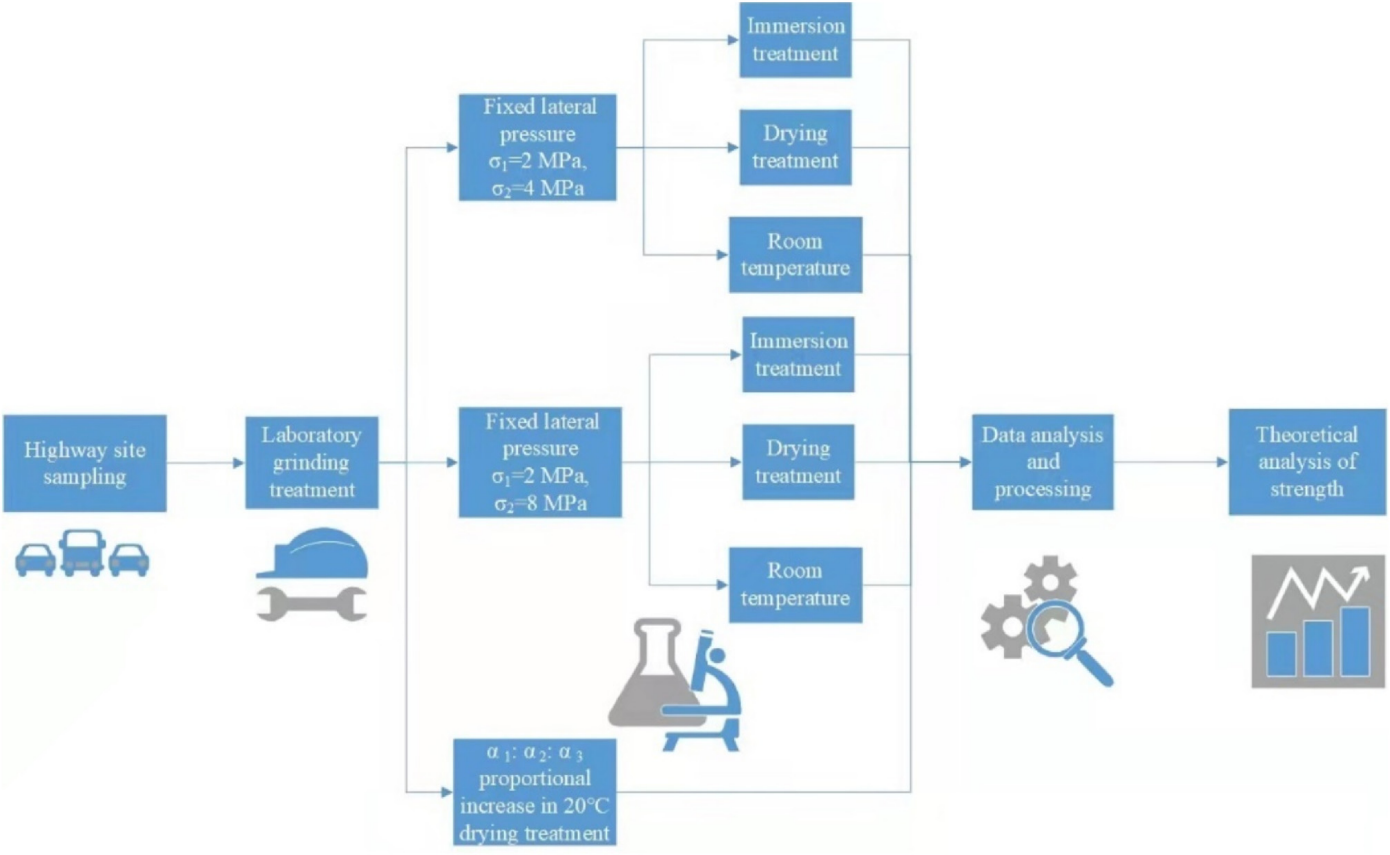
Flow chart of methodologies.

To study the influence of different intermediate principal stresses on the triaxial strength of asphalt mixtures, referring to the true triaxial test scheme of rock and concrete by Guo et al. [44], two triaxial loading modes are selected in this study. One is fixed lateral pressure, and two different test lateral pressures of *σ*_1_ = 2 MPa, *σ*_2_ = 4 MPa and *σ*_1_ = 2 MPa, *σ*_2_ = 8 MPa are applied while applying *σ*_3_. The other is that the lateral pressure increases proportionally, and the design stress ratios *α* = α_1_:α_2_:α_3_ are 0.1:0.25:1; 0.1:0.5:1; 0.1:0.75:1 and 0.1:1:1. Since the asphalt mixtures are visco-elastic materials, their failure behaviour can be influence by the loading rate [45, 46, 47]. Under the combined action of different temperature and loading rate, the fracture energy of asphalt mixture will change greatly [48]. Too fast loading rate may lead to rapid failure of the specimen, which is not conducive to recording its failure process. According to the triaxial test of Guan et al. [36] *σ*_3_ appropriate loading rate is conducive to fully show the whole process of specimen failure. Then, *σ*_3_ was increased by 0.1 MPa/s until the samples were broken. The experiments were conducted using the stress control mode. For better understanding, the flow chart of methodologies is shown in Figure 5.

## 3 Results and discussion

### 3.1 Failure form characteristics

**Figure 6:**
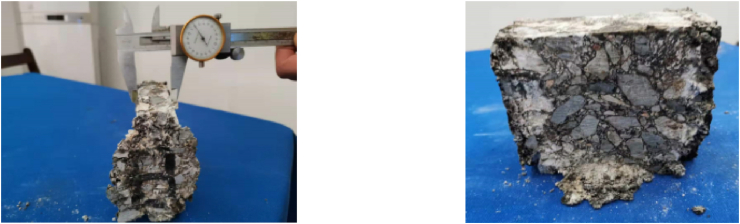
Failure surface of triaxial loading specimen.

Under triaxial compression, the asphalt mixture specimen does not show obvious splitting failure or spalling failure as a whole and shows obvious strain hardening characteristics. With increasing stress, the specimen density gradually increases until the direction of ε_3_ reaches 40%. There are still no cracks penetrating the specimen, but there are obvious shear plane cracks, as shown in Figure 6. This shows that the asphalt mixture specimen was damaged under a certain load value before the end of the test.

### 3.2 Axial stress–strain relationship

**Figure 7:**
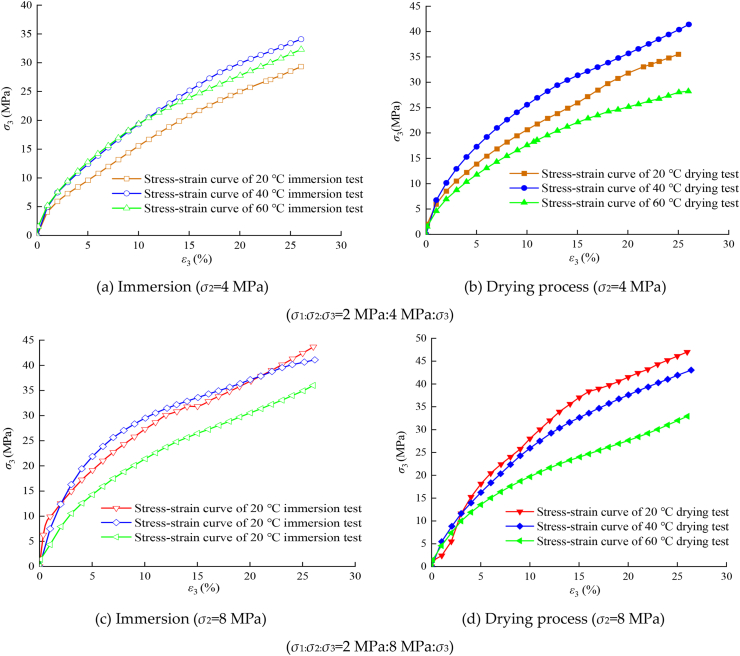
Axial stress–strain curve.

Taking the fixed lateral pressure loading as an example, the axial stress–strain relationship curve under immersion and drying environments is shown in Figure 7. It can be seen that there is an obvious peak point different from that under uniaxial compression. The lateral stress prevents expansion deformation under large axial compression and improves the stress level of cracks in the specimen.

### 3.3 Damage pressure

**Figure 8:**
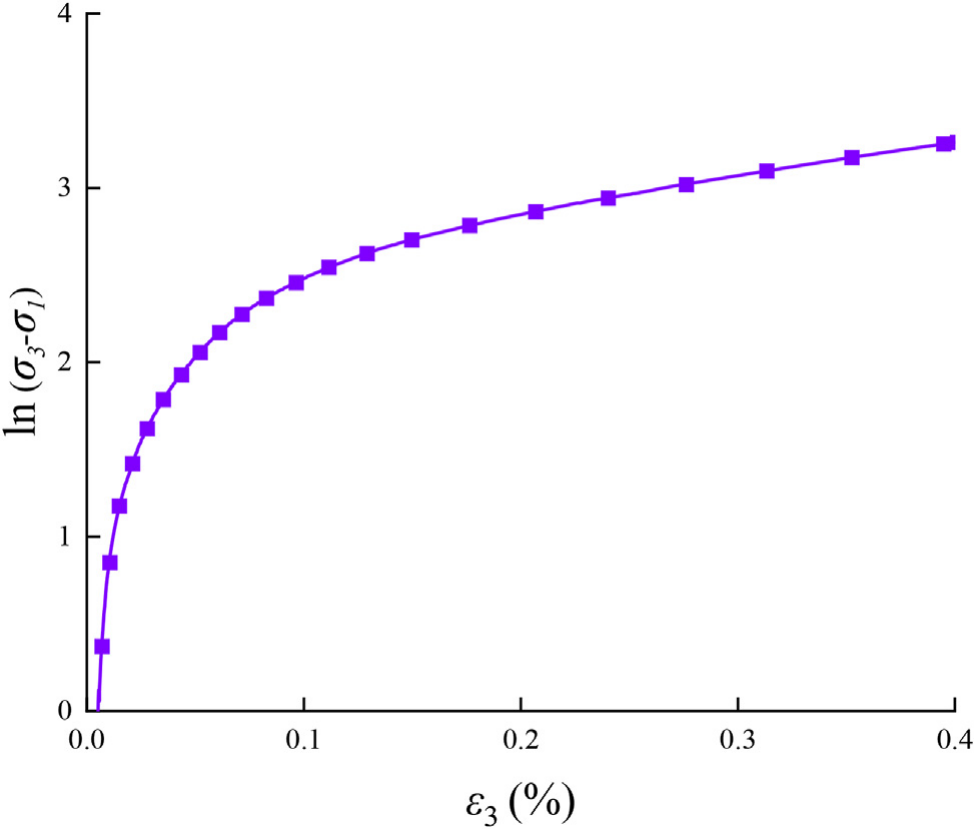
ln (*σ*_3_-*σ*_1_) ∼ *ε*_3_ relation curve.

There are no detailed provisions on the value-taking method of the axial compressive strength of triaxial tests in the existing mechanically related test codes and regulations. In previous studies, the compressive strength of rock or concrete and other materials under similar conditions can be determined by mechanical indices such as logarithmic curves and rate curves of stress and strain [49]. The logarithm of the shear stress under the drying environment of 40 °C (*σ*_1_: *σ*_2_: *σ*_3_ = 2 MPa:4 MPa:*σ*_3_) can be obtained, as shown in Figure 8.

**Figure 9:**
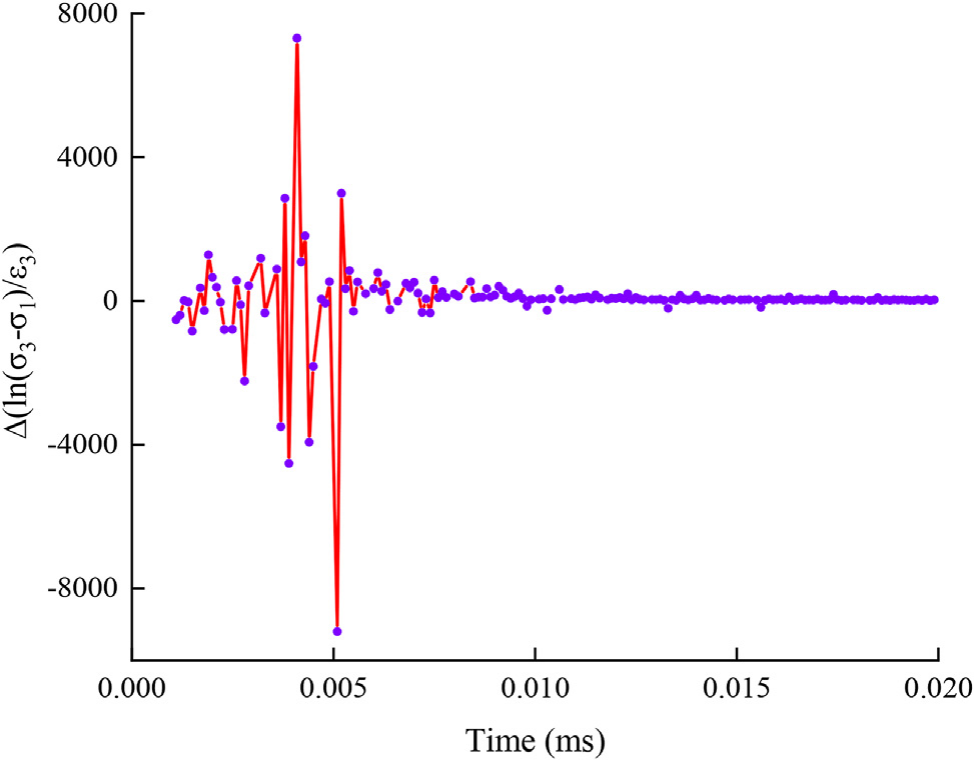
ln (*σ*_3_-*σ*_1_) ∼ *ε*_3_ Change rate diagram.

For ln ($\sigma_{1}$: $\sigma_{2}$: $\sigma_{3}$) extracting the relative instantaneous change rate ε_3_, the ∼ change rate curve can be obtained, as shown in Figure 9. The change rate of the shear stress shows a gradual decreasing trend. The absolute value of the change rate is finally stable at approximately 1, and the asphalt mixture enters the strain hardening stage.

The asphalt mixture has undergone large deformation in the yield stage. Therefore, the moment when the change rate ln ($\sigma_{3\;}$-$\;\sigma_{1}$) enters 1 is regarded as failure, and the corresponding axial stress is the failure stress of the specimen. The failure stress value of the specimen is defined as its triaxial compressive strength, and the corresponding axial strain value is defined as the yield strain. In different environments, repeat the test for three times in each test environment, and all test data are listed in Table 5. The dispersion coefficient of the test results is mostly less than 5%, which proves that the test results can accurately reflect the real situation by excluding the influence caused by the test error.

**Table 5:** Failure stress data of asphalt mixture.

*σ*_1:_*σ*_2_	Test environment			Compressive strength under different drying temperature/MPa			Standard deviation values	Limit of error	Discrete coefficient
				*σ*_3_-01	*σ*_3_-02	*σ*_3_-03			
2:4	Room temperature			32.006	30.44	32.804	1.20	-5.36%–1.99%	3.79%
	Drying temperatures		20 °C	29.48	29.538	33.472	2.29	-6.79%–5.86%	7.42%
			40 °C	25.404	26.747	26.299	0.68	-3.72%–1.37%	2.61%
			60 °C	18.046	17.485	19.219	0.88	-5.79%–3.55%	4.85%
	Immersion temperatures		20 °C	28.078	27.146	27.306	0.50	-1.94%–1.43%	1.81%
			40 °C	22.747	23.522	23.241	0.39	-2.4%–0.93%	1.69%
			60 °C	24.402	23.582	23.776	0.43	-2.02%–1.38%	1.79%
2:8	Room temperature			37.068	36.49	37.112	0.35	-1.04%–0.28%	0.94%
	Drying temperatures	20 °C		36.854	36.622	33.144	2.08	-8.58%–1.66%	5.85%
		40 °C		34.004	34.418	32.348	1.10	-4.77%–1.33%	3.26%
		60 °C		24.781	25.955	26.934	1.08	-5.65%–2.55%	4.16%
	Immersion temperatures	20 °C		30.307	32.038	29.905	1.13	-2.75%–4.19%	3.69%
		40 °C		35.421	35.727	32.262	1.92	-6.41%–3.65%	5.57%
		60 °C		28.489	26.99	27.591	0.75	-2.53%–2.89%	2.72%

### 3.4 The influence of hygrothermal environments and intermediate principal stress on the compressive strength and yield strain

When the asphalt mixture is loaded under fixed lateral pressure, its compressive strength and yield strain under different drying temperatures and different immersion temperatures are listed in Tables 6, 7, 8, and 9. To compare the strength of the asphalt mixture in a common environment, the test was also performed at room temperature (20 °C).

**Table 6:** Compressive strength of asphalt concrete under different drying temperatures.

*σ*_1:_*σ*_2_	Compressive strength under different drying temperature/MPa			
	Room temperature	20 °C	40 °C	60 °C
0 (uniaxial)	3.79 (−/−)	3.75 (-/1.05)	2.79 (-/26.38)	2.35 (-/37.94)
2:4	31.75 (737.73/-)	30.83 (722.13/2.89)	26.15 (837.27/17.63)	18.25 (676.59/42.51)
2:8	36.89 (873.75/-)	35.54 (847.73/3.66)	33.59 (1103.94/8.94)	25.89 (1001.70/29.81)

Note: the number before “/” represents the increase (%) in damage stress compared with that under uniaxial compression, while the number after “/” represents the decrease (%) in damage stress at room temperature.

**Table 7:** Yield strain of asphalt concrete under different drying temperatures.

*σ*_1:_*σ*_2_	Yield strain under different drying temperature/%			
	Room temperature	20 °C	40 °C	60 °C
0 (uniaxial)	5.43 (−/−)	5.59 (-/2.94)	5.09 (-/6.26)	4.50 (-/17.12)
2:4	20.15 (271.08/-)	19.01 (240.07/5.65)	15.03 (195.28/25.41)	10.67 (137.11/47.05)
2:8	15.89 (192.63/-)	14.09 (152.06/11.32)	16.00 (214.43/0.69)	13.63 (202.89/14.22)

Note: the number before “/” represents the increase (%) in damage strain compared with that under uniaxial compression, while the number after “/” represents the decrease (%) in damage strain at room temperature.

**Table 8:** Compressive strength of asphalt concrete under different immersion temperatures.

*σ*_1:_*σ*_2_	Compressive strength under different immersion temperature/MPa			
	Room temperature	20 °C	40 °C	60 °C
0 (uniaxial)	3.79 (−/−)	3.87 (-/2.11)	3.52 (-/7.12)	2.76 (-/27.17)
2:4	31.75 (737.73/-)	27.51 (610.85/13.35)	23.17 (558.23/27.02)	23.92 (676.59/24.66)
2:8	36.89 (873.75/-)	30.75 (694.57/16.64)	34.47 (879.26/6.56)	27.69 (903.26/24.93)

Note: the number before “/” represents the increase (%) in damage stress compared with that under uniaxial compression, while the number after “/” represents the decrease (%) in damage stress at room temperature.

**Table 9:** Yield strain of asphalt concrete under different immersion temperatures.

*σ*_1:_*σ*_2_	Yield strain under different immersion temperature/%			
	Room temperature	20 °C	40 °C	60 °C
0 (uniaxial)	5.43 (−/−)	5.59 (-/2.94)	5.09 (-/6.26)	4.50 (-/17.12)
2:4	20.15 (71.08/-)	16.29 (191.41/19.15)	13.26 (160.51/34.19)	15.79 (250.89/21.63)
2:8	15.89 (192.63/-)	13.91 (148.83/12.46)	16.25 (219.25/2.26)	15.27 (239.33/3.9)

Note: the number before “/” represents the increase (%) in damage strain compared with that under uniaxial compression, while the number after “/” represents the decrease (%) in damage strain at room temperature.

Table 6 shows that under drying treatment, the compressive strength of the asphalt mixture decreases with increasing ambient temperature; the reduction amplitude also increases with increasing temperature, and the amplitude reaches its maximum at 60 °C. When *σ*_2_ increases from 4 MPa to 8 MPa, compared with uniaxial compression, the increase percentage of damage stress decreased from 42.51% to 29.81%, indicating that the increase in intermediate principal stress will increase the absolute value of failure stress and inhibit the decrease in amplitude caused by the temperature increase at 40 °C, and the inhibition effect reaches the maximum at this temperature.

Table 7 shows that under drying treatment, the yield strain of asphalt concrete will decrease with increasing ambient temperature, the decrease amplitude will also increase with increasing temperature, and the decrease amplitude is the highest at 60 °C. At 60 °C, it increases from 4 MPa to 8 MPa, compared with uniaxial compression, the increase percentage of yield strain decreased from 47.05% to 14.22% at room temperature, which indicates that the increase in intermediate principal stress will increase the yield strain and restrain the influence of the temperature increase.

Table 8 shows that under water immersion treatment, the compressive strength of the asphalt mixture decreases with increasing ambient temperature, and the reduction amplitude increases with increasing temperature under uniaxial loading and gradually decreases with increasing temperature under triaxial loading. When *σ*_2_ increases from 4 MPa to 8 MPa, compared with uniaxial compression, the increase percentage of damage stress decreased from 27.02% to 6.56% and 24.66%–24.93% at 40 °C and 60 °C respectively. At 40 °C, the difference of compressive strength between the two experimental groups reached the maximum, indicating that the increase of intermediate principal stress weakens the effect of temperature on compressive strength, which reaches the maximum at 40 °C.

Table 9 shows that under water immersion treatment, the yield strain of asphalt concrete decreases with the increase of ambient temperature, and the reduction amplitude will increase with the increase of temperature under uniaxial loading and gradually decrease with the increase of temperature under triaxial loading. When increasing from 4 MPa to 8 MPa, compared with uniaxial compression, the increase percentage of yield strain decreased from 34.19% to 2.26% and 21.63%–3.90% at 40 °C and 60 °C respectively. At 60 °C, the yield strain of the two experimental groups has little difference, indicating that the increase in intermediate principal stress will weaken the effect of temperature on strain.

Asphalt mixture consists of aggregate, pores and asphalt colloid. Among them, asphalt colloid plays a role in bonding aggregate and supporting strength. Asphalt mortar is highly sensitive to temperature and humidity. Under drying, the adhesion between asphalt mortar and aggregate decreases, the degree of freedom increases, and the resistance to deformation decreases under load. It is reflected in the macro mechanical properties, that is, the triaxial compressive strength, yield strain and elastic modulus of AC-25C asphalt mixture decrease with the increase of drying temperature. When the lateral pressure is increased, the softening effect of asphalt is limited, and the strength reduction effect caused by temperature rise is restrained to a certain extent. That is, when the intermediate principal stress is increased, the amplitude of stress-strain decrease caused by temperature rise also begins to decrease.

When the temperature and humidity are coupled, water immersion in the asphalt mixture also reduces the adhesion of the mixture, making the triaxial compressive strength, yield strain and elastic modulus of the asphalt mixture decrease with the increase of immersion temperature. However, unlike drying, the water in the gap plays a certain role in resisting the material deformation, and the increase of side pressure inhibits the outflow of water in the mixture, The deformation resistance of the specimen is improved to a certain extent, and this resistance reaches the maximum when the immersion temperature is 40 °C. It is shown in the macroscopic mechanical properties, that is, when the intermediate principal stress is increased, the amplitude of stress-strain decrease caused by heating increases first and then decreases.

In previous studies, the compressive strength of rock or concrete and other materials under similar conditions can be determined by mechanical indices such as logarithmic curves and rate curves of stress and strain [44].

## 4 Failure criterion of asphalt concrete in hot and humid environments

The strength and failure criterion of materials is a common and important scientific problem. The existing research on the strength of asphalt concrete mostly adopts the octahedral shear stress strength theory, in which the reason for material failure is the stress on the octahedral surface reaching the limit value [10]. The mathematical expression is used to fit the material failure envelope surface, which is widely used in concrete, rock, and other materials. The traditional method to determine the theoretical parameters of octahedral strength and shear stress is mainly determined by mathematical fitting of test data, and the test results can be verified at the same time. The existing research results on the octahedral criterion of asphalt mixtures are obtained by confining pressure triaxial tests. The strength criterion obtained has certain limitations because it cannot accurately reflect the role of intermediate principal stress [41].

According to the experimental data in the previous section, the triaxial compressive strength of materials changes greatly under different damp and hot environments. Combined with the true triaxial compression test data and based on the octahedral shear stress strength theory, this paper establishes the failure criterion under damp and hot environments, improves it, and adds the temperature humidity coefficient k to reflect the damp and heat effects. An improved failure criterion of asphalt concrete in hot and humid environments is estab-lished.

### 4.1 Failure criterion of asphalt mixtures based on octahedral shear stress strength theory

In the principal stress space, the three-dimensional diagram of the failure envelope surface is often not easy to draw and express; thus, it is often expressed by the plane graph on the tension compression meridional plane. The intersection of this plane and the failure envelope is called the tension and compression meridian, respectively. By analysing the data points of the true triaxial compression test, the compression meridian equation of the asphalt concrete failure criterion can be fitted in the form of a functional equation, and the relationship between relative normal stress and relative shear stress of asphalt concrete octahedron is established as follows [50, 51]:

(1) $$\frac{\tau_{\text{oct}}}{f_{\text{c}}}=A{\left(\frac{\sigma_{\text{oct}}}{f_{\text{c}}}\right)}^{2}+B\left(\frac{\sigma_{\text{oct}}}{f_{\text{c}}}\right)+C$$

where coefficients A, B and C are the material parameters and uniaxial compressive strength of asphalt concrete. $\left(\frac{\sigma_{otc}}{f_{c}}\right)$ and $\left(\frac{\tau_{otc}}{f_{c}}\right)$ are the values of normal stress and shear stress on the compression meridian. $\sigma_{otc}$ and $\tau_{otc}$ are the octahedral normal stress and shear stress and are calculated by the equations below [50].

(2) $$\sigma_{otc}=\frac{1}{3}\left(\sigma_{1}+\sigma_{2}+\sigma_{3}\right)$$

(3) $$\tau_{otc}=\frac{1}{3}\sqrt{{\left(\sigma_{1}-\sigma_{2}\right)}^{2}+{\left(\sigma_{2}-\sigma_{3}\right)}^{2}+{\left(\sigma_{3}-\sigma_{1}\right)}^{2}}$$

where $\sigma_{1}\;$ is the maximum principal stress, $\sigma_{2}$ is the intermediate principal stress, and $\sigma_{3}$ is the minimum principal stress.

#### 4.1.1 Characteristics of the octahedral pressure meridian under different loading modes in a constant environment

According to the test data in Tables 6 and 8, the octahedral normal stress $\sigma_{otc}$ and shear stress $\tau_{otc}$, normal stress $\left(\frac{\sigma_{otc}}{f_{c}}\right)$ and shear stress $\left(\frac{\tau_{otc}}{f_{c}}\right)$ on the compression meridian of the AC-25C asphalt mixture under a drying environment of 20 °C are calculated by Eqs. (2) and (3), respectively. The calculated results are shown in Table 10.

**Table 10:** Octahedral stress parameters.

Experimental environments	$\left(\sigma_{1},\sigma_{2},\sigma_{3}\right)$ (MPa)	$\sigma_{otc}$	$\tau_{otc}$	$\frac{\sigma_{otc}}{f_{c}}$	$\frac{\tau_{otc}}{f_{c}}$
20 °C drying	(2.00, 4.00, 30.83)	12.278	13.15	3.27	3.51
	(2.00, 8.00, 35.54)	15.182	14.60	4.05	3.89

**Figure 10:**
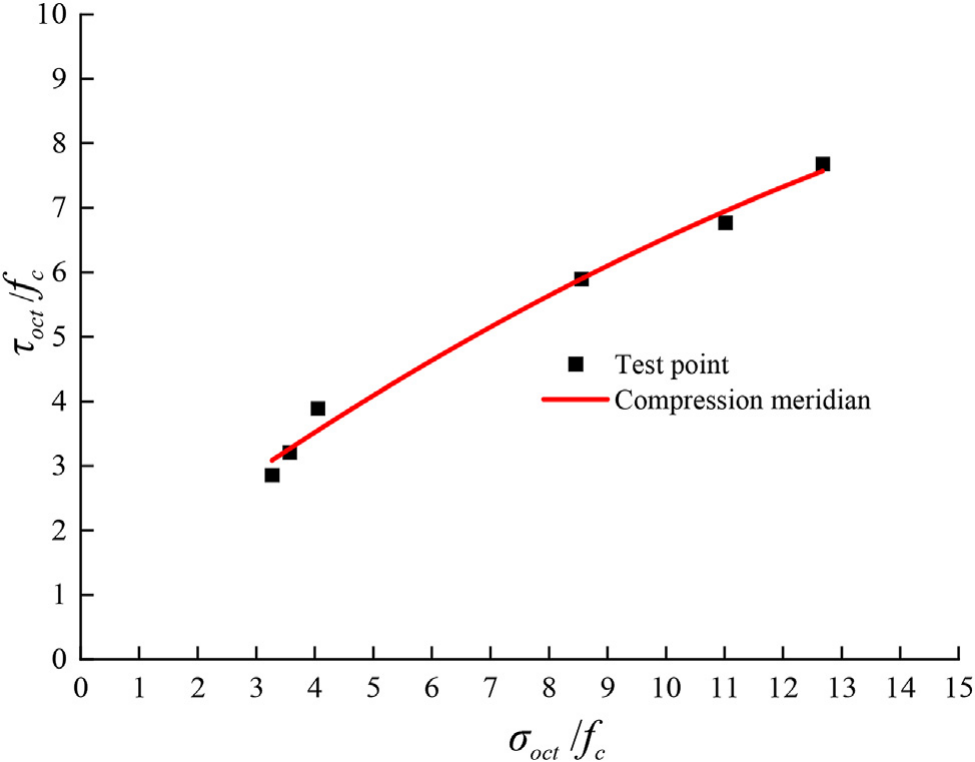
Compressive meridians in the 20 °C drying test.

Eq. (1) and Table 10 are used to perform least squares regression analysis on the test data of two different loading forms under the 20 °C drying environment, and the failure criterion of the asphalt mixture under the 20 °C drying environment can be obtained. The material parameter values are shown in Table 11, and the pressure meridian is shown in Figure 10.

**Table 11:** Break criteria parameter list.

A	B	C	*R*^2^
-0.01286	-0.70796	0.6955	0.9907

Figure 10 shows that the strength criterion curve is smooth and continuous, and the octahedral shear stress value on the compression meridian increases with increasing absolute value of the octahedral relative normal stress. The established true triaxial failure criterion model is in good agreement with the test data. With an increase in the absolute value of the relative normal stress, the failure curve slowly increases, and the slope gradually decreases. The curve has a horizontal development trend.

#### 4.1.2 Characteristics of the octahedral pressure meridian under fixed side pressure loading in hygrothermal environments

**Figure 11:**
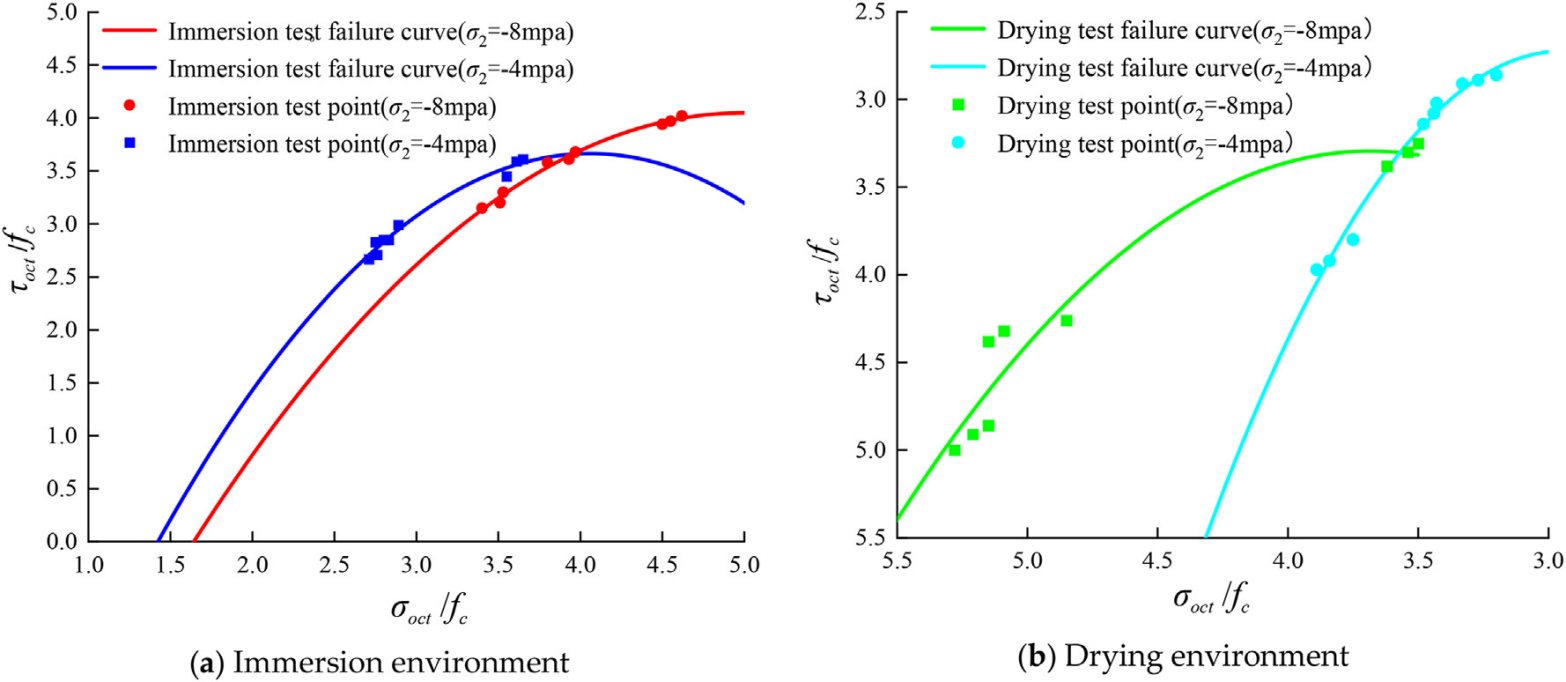
Compressive meridians in a hygrothermal environment considering the intermediate principal stress effect.

According to the experimental data, the normal stress and shear stress values on the compression meridian of AC-25C asphalt concrete under fixed lateral pressure loading in different humid and hot environments are calculated. The experimental data under each loading mode are fitted through Eq. (1), and the octahedral stress space failure envelope curve under different lateral pressures is obtained. The comparison with the test results is shown in Figure 11.

Figure 11 shows that the strength criterion curve under the immersion environment is continuous, smooth and convex. With the increase in the intermediate principal stress, the extreme point of the curve gradually moves outward, indicating that the increase in the intermediate principal stress will increase the triaxial compressive strength of the asphalt concrete under the immersion state. Under the drying environment, the failure curve is continuous, smooth and concave. With the increase in intermediate principal stress, the extreme point of the curve gradually moves outward, indicating that under the drying state, the increase in intermediate principal stress will also increase the triaxial compressive strength of asphalt concrete. According to the abovementioned results, it can be concluded that the octahedral strength theory has good applicability for the establishment of strength criteria of asphalt mixtures under different wet and hot environments.

### 4.2 Failure criterion of octahedral shear stress theory considering the effects of hygrothermal environments

**Figure 12:**
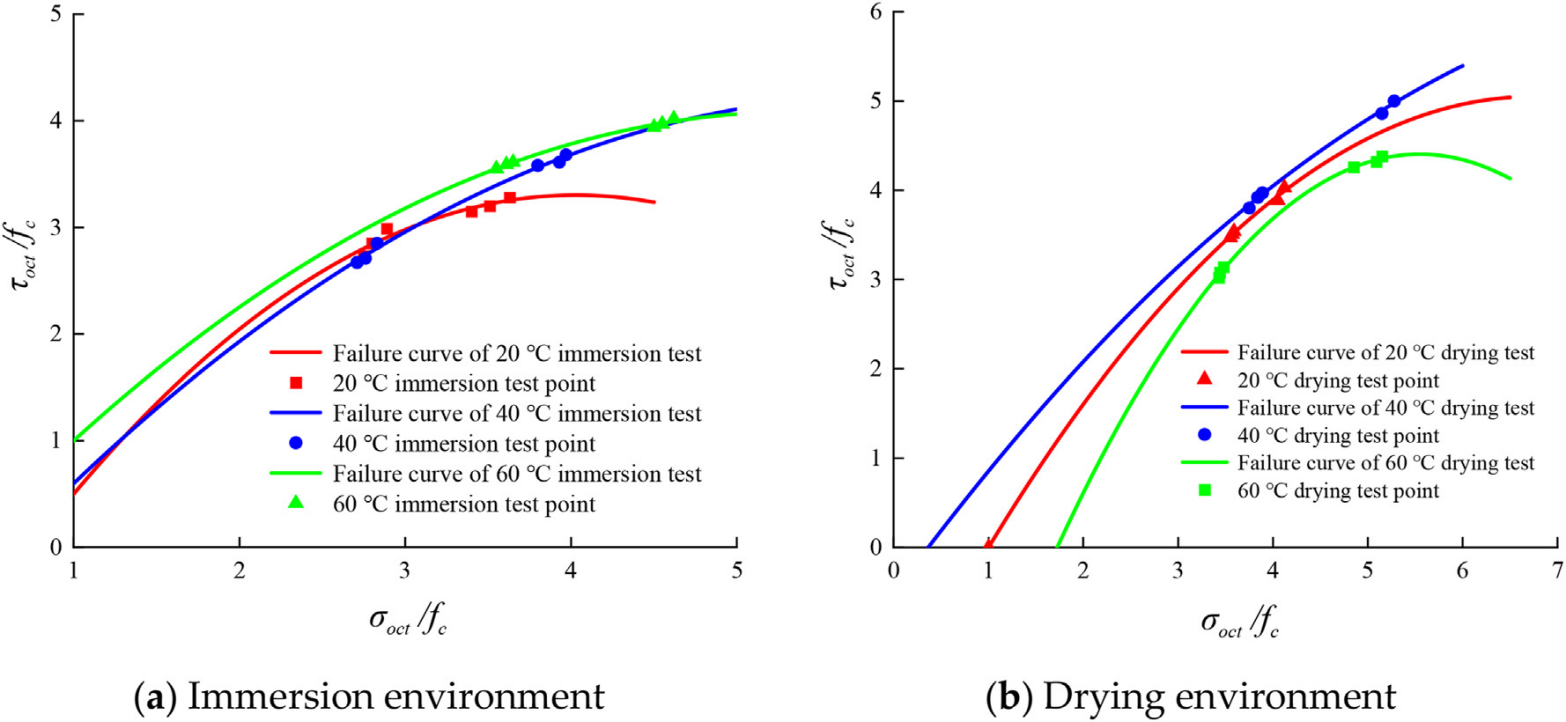
Compressive meridians in a hygrothermal environment considering the temperature effect.

According to the experimental data, the normal stress and shear stress values on the compression meridian of AC-25C asphalt concrete under fixed lateral pressure loading in different humid and hot environments are calculated. The experimental data under each loading mode are fitted through Eq. (1), and the octahedral stress space failure envelope curve under different lateral pressures is obtained. The comparison with the test results is shown in Figure 12.

Figure 12 shows that under the immersion environment, the failure curve of asphalt concrete is continuous, smooth and convex. With increasing immersion temperature, the extreme point of the curve gradually moves outward, indicating that under the immersion state, the increase in temperature will increase the triaxial compressive strength of asphalt concrete. The failure curve under a drying environment is continuous, smooth and convex. With the increase in intermediate principal stress, the extreme point of the curve first increases and then decreases, indicating that in the range of 20–60 °C, the strength of asphalt concrete reaches its maximum at 40 °C and the envelope range of the failure curve reaches its maximum.

The strength of the asphalt mixture is closely related to the temperature and humidity of its environment, and its influence should also be reflected in the tension compression meridian equation. Figure 12 shows that the failure curves at different temperatures are smooth and convex. To facilitate engineering applications, the coefficient of strength criterion equation is simplified in this study.

**Figure 13:**
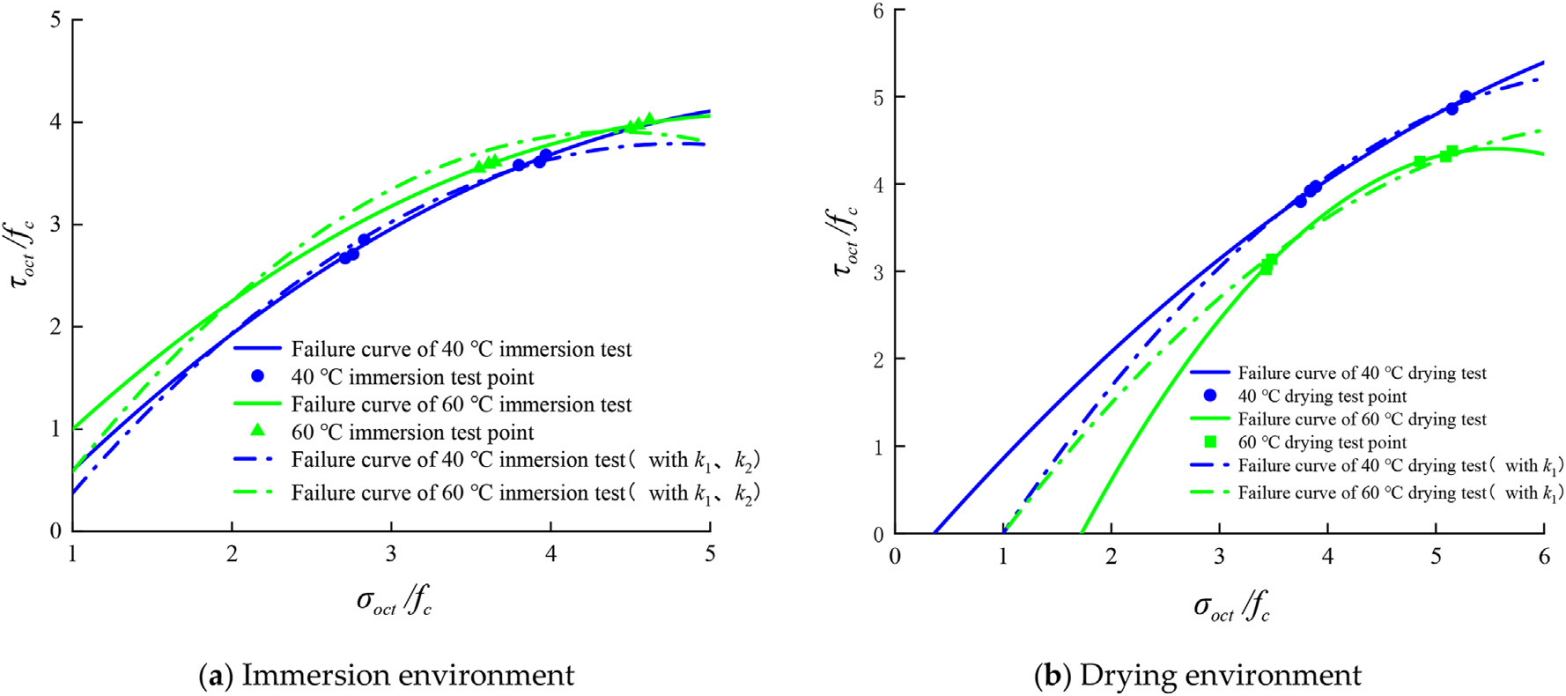
Comparison of failure criterion meridians with damp heat parameters and test curves.

Referring to the treatment methods of influencing factors such as specimen size and temperature in the study of concrete triaxial strength law by Ren et al. [51], this study improves Eq. (3) by introducing the temperature humidity coefficient *k* and considering the influence of temperature and humidity on strength and puts forward a new failure criterion including damp heat parameters as shown in Eq. (4).

(4) $$\frac{\tau_{oct}}{f_{c}}=k\left[A^{t}{\left(\frac{\sigma_{otc}}{f_{c}}\right)}^{2}+B^{t}{\left(\frac{\sigma_{otc}}{f_{c}}\right)}^{2}+C^{t}\right]$$

where, $A^{t}$, $B^{t}$ , and $C^{t}$ are, respectively, the coefficients of the quadratic term, primary term and constant term of the failure curve obtained by fitting the test data under the immersion and drying environment at 20 °C, and the three coefficients have fixed values. The temperature humidity coefficient *k* is obtained by fitting the experimental data. The parameter values of *k* under different damp and heat environments are listed in Tables 12 and 13. The comparison between the failure criterion meridian with damp and heat parameters and the test curve is shown in Figure 13.

**Table 12:** Values of relevant parameters of the damage ratio of the immersion treatment test group.

	$A^{t}$	$B^{t}$	$C^{t}$	k	*R*^2^
20 °C immersion	-0.305	-2.460	-1.655	1	0.990
40 °C immersion	-0.305	-2.460	-1.655	1.05	0.780
60 °C immersion	-0.305	-2.460	-1.655	1.16	0.728

**Table 13:** Values of relevant parameters of the damage ratio of the drying treatment test group.

	$A^{t}$	$B^{t}$	$C^{t}$	*k*	*R*^2^
20 °C drying	-0.152	-2.060	-1.907	1	0.998
40 °C drying	-0.152	-2.060	-1.907	1.06	0.904
60 °C drying	-0.152	-2.060	-1.907	0.93	0.915

Figure 13 shows that the strength criterion with temperature coefficient *k* proposed in this study can better describe the triaxial failure curve under different humid and hot environments within a certain temperature range and shows the change trend of the failure curve. In the immersion environment, with increasing temperature, the value of *k* gradually increases, and the envelope surface of the failure curve also gradually increases. In the drying environment, with increasing temperature, the *k* value first increases and then decreases, which is reflected in the failure curve; that is, the envelope surface first expands and then decreases, which is also consistent with the abovementioned test law.

With the increase of temperature, the adhesion of asphalt colloid in asphalt mixture decreases. At this time, the addition of water will accelerate the aging of asphalt and reduce the strength of asphalt mixture [50]. With the increase of time, this aging effect will show a nonlinear growth trend, but in the short term, compared with the intermediate principal stress, this aging effect has less impact on the strength of asphalt mixture. The treatment time of temperature in this study is 24 h so the asphalt is still in the primary stage of aging, and the aging effect is not significant. At this moment, the intermediate principal stress is the main factor affecting the strength of asphalt mixture. Moreover, in the octahedral stress space, all relative values are dimensionless, and the gap between the two different influencing factors is enlarged, resulting in no obvious temperature effect in Figures 12 and 13.

The performed analysis shows that the true triaxial failure criterion of the asphalt mixture considering the temperature coefficient *k* proposed in this study fits the test results well in a certain temperature range. Based on the test results at 20 °C, the triaxial compression failure curve trend of asphalt mixtures under different humid and hot environments can be predicted, and the calculation accuracy is generally good. The method has good reference significance for optimizing the test design and studying the triaxial failure strength of asphalt mixtures under different hygrothermal environments.

## 5 Conclusions

1. The AC-25C asphalt mixture under triaxial compression shows significant strain hardening characteristics. Because there is no direct peak point or falling section in the stress–strain curve, it can be judged by mechanical indices such as the logarithmic curve and rate curve of stress and strain. The performed research shows that under true triaxial compression, the change rate of the shear stress of the asphalt mixture specimen shows a decreasing trend. The absolute value of the change rate is finally stable at approximately 1. At this time, it can be regarded as failure, and its failure point can be determined.
2. Under the true triaxial test condition of constant lateral pressure, the lateral stress of the asphalt mixture remains stable, and the lateral expansion deformation begins with the increase in axial stress. The constant lateral stress hinders the early expansion deformation of the specimen, limits the rapid development of later expansion deformation, improves the maximum volume compression value of the specimen, enhances the crack resistance of the specimen, and improves the axial compression strength shrinkage capacity. The increase in the triaxial compressive strength of the asphalt mixture with the increase in the medium principal stress ratio is *σ*_2_/*σ*_3_, and the strength growth rate reaches the maximum in the range of *σ*_2_/*σ*_3_ = 0.25–0.5.
3. The three-dimensional failure criteria under hygrothermal environments of AC-25C asphalt mixtures are presented for the first time based on combination strength tests. Based on the octahedral stress space, the failure criterion considering the temperature humidity coefficient k is proposed in this study. There is a multivariate linear relationship between the octahedral shear strength, octahedral normal strength, and temperature humidity coefficient *k.* The proposed method can determine the failure criteria for asphalt mixtures with different gradations, temperatures, and humidities and provide suggestions and references for engineering applications.

## Declarations

### Author contribution statement

Wang Pan: Performed the experiments; Analyzed and interpreted the data; Contributed reagents, materials, analysis tools or data; Wrote the paper.

Mohamed Elchalakani, Yiming Zhou, Shi-tao Yan, Shuang-bei Li: Conceived and designed the experiments; Analyzed and interpreted the data; Wrote the paper.

### Funding statement

This research was supported by grants awarded by the National Natural Science Foundation of China (no. 51878186) and the Science and Technology Major Project of Guangxi Province (no. AA18118029).

### Data availability statement

Data will be made available on request.

### Declaration of interest’s statement

The authors declare no conflict of interest.

### Additional information

No additional information is available for this paper.
